# Exploring the Hearing Improvement and Parental Stress in Children with Hearing Loss Using Hearing Aids or Cochlear Implants

**DOI:** 10.3390/jcm14010002

**Published:** 2024-12-24

**Authors:** Daniele Portelli, Clara Lombardo, Sabrina Loteta, Cosimo Galletti, Carmela Azielli, Francesco Ciodaro, Carmela Mento, M’Hammed Aguennouz, Gabriella Di Rosa, Angela Alibrandi, Giuseppe Alberti

**Affiliations:** 1 Unit of Otorhinolaryngology, Department of Adult and Development Age Human Pathology “Gaetano Barresi”, University of Messina, 98122 Messina, Italy; daniele.portelli09@gmail.com (D.P.); lotetas@unime.it (S.L.); aziellic@unime.it (C.A.); dottfciodaro@alice.it (F.C.); galberti@unime.it (G.A.); 2 Department of “Scienze della Salute”, University of “Magna Graecia”, 88100 Catanzaro, Italy; clara.lombardo@unicz.it; 3 Faculty of Medicine and Surgery, Kore University of Enna, 94100 Enna, Italy; cosimo.galletti01@unikore.it; 4 Department of Biomedical and Dental Sciences and Morphofunctional Imaging, University of Messina, 98122 Messina, Italy; gabriella.dirosa@unime.it; 5 Department of Clinical and Experimental Medicine, University of Messina, 98122 Messina, Italy; aguenoz.mhommed@unime.it; 6 Unit of Statistical and Mathematical Sciences, Department of Economics, University of Messina, 98122 Messina, Italy; aalibrandi@unime.it

**Keywords:** cochlear implants, hearing aids, parental stress, auditory performance, congenital hearing loss

## Abstract

**Objectives:** This study aims to describe the stress levels experienced by parents of children with hearing loss who use conventional hearing aids or cochlear implants, and to assess the correlation between parental stress and the auditory skills acquired by the children. **Methods:** The study was conducted at the Policlinic “Gaetano Martino” in Messina, evaluating data from 42 pairs of parents of children using hearing aids or cochlear implants. Parents completed the LittlEARS Auditory Questionnaire (LEAQ) and the Parental Stress Scale (PSS) 18 months after the initial device (hearing aid or cochlear implant) had been activated. Additionally, information was collected regarding the presence of peripartum issues (including preterm birth) or associated conditions, congenital hearing loss, the total number of children in the family, and the number of children with hearing loss in the family. **Results:** Significant differences were found in the months to effective stimulation (*p* = 0.026), the age of the children at the time of the survey (*p* = 0.024) and the PSS score (*p* = 0.029). Univariate and multivariate logistic regression revealed significant correlations between LEAQ scores and both the months to effective stimulation and the age of the children at the time of the survey; univariate and multivariate linear regression revealed significant correlations between PSS scores and the type of device, months to effective stimulation, age of the children at the time of the survey, peripartum issues, and the number of children. A Spearman correlation showed a positive relationship between LEAQ and age of the children at the time of the survey, and a negative correlation between the PSS scores and the age of the children at the time of the survey. **Conclusions:** Parents of children with cochlear implants reported higher stress levels than those with children using hearing aids, although auditory performance was comparable between groups. Improved auditory performance was associated with reduced parental stress. The PSS and LEAQ are effective tools used in clinical practice for assessing parental stress and tracking auditory recovery, respectively.

## 1. Introduction

The World Health Organization (WHO), in its last report, estimated that approximately 1.5 billion people worldwide suffer from hearing loss, with 432 million adults and 34 million children requiring rehabilitation to address their hearing issues [1]. The prevalence of bilateral moderate to severe hearing impairment varies from 1–3 per 1000 normal newborns to about 2–4 per 1000 in high-risk newborns [2].

Hearing is crucial in children for language acquisition and cognitive development; it facilitates communication throughout the developmental stages and enhances social interactions [3]. Failure to identify and address hearing loss, especially in cases of congenital deafness, poses a significant obstacle to a child’s development, education, and social integration [4].

Another important consideration concerns the timing of identifying hearing loss and the age of intervention to address it; children who receive early diagnosis and rehabilitation will have better outcomes compared to those with delayed intervention [4,5,6]. Moreover, it has been demonstrated that children with congenital deafness who receive appropriate intervention within the first six months of age will have auditory and language development at 5 years on par with their normal-hearing counterparts [4,7].

Today, hearing aids, bone conduction implants and cochlear implants are the most widely used devices in the treatment of irreversible hearing loss to restore functional listening quality [1,2,3,5,7,8].

They represent a significant technological advancement in the rehabilitative treatment of children with congenital deafness, enabling them to acquire language and excellent verbal skills [1,5]. This, of course, is crucial for ensuring a good process of socialization, education, and self-confidence [4,9].

However, it is important to note that outcomes also depend on the caregivers of these individuals, including healthcare professionals and family members [10]. Indeed, factors influencing the functional recovery of auditory–verbal skills and, ultimately, the child’s overall functional outcomes include the age at which the hearing rehabilitation is commenced, the duration of auditory deprivation, and, crucially, a family environment predisposed to providing adequate assistance to the child’s various needs [4,5,6,9,10].

For instance, it has been demonstrated that auditory skills and communicative abilities may not be adequate in children even after receiving a cochlear implant [10]. Additionally, the pre-implantation and post-surgical rehabilitation process can represent a source of stress for parents due to the challenges they must navigate [10].

It has also been demonstrated that parents of children with hearing loss who receive cochlear implants experience greater psychological stress compared to parents of children with hearing aids [10,11,12].

The aim of this study is to describe the level of stress experienced by parents of children with hearing loss who use conventional hearing aids or cochlear implants, and to assess its correlation with the auditory skills acquired by the child. In this regard, a comparative analysis was conducted among the parents of children treated at our tertiary center using self-assessment questionnaires as evaluation scales.

## 2. Materials and Methods

### 2.1. Participants

The study was conducted at the tertiary referral center of Policlinic “Gaetano Martino” in Messina. Data from 42 couples of parents (each couple consisting of a mother and father, either being the primary caregiver) of children using conventional hearing aids or cochlear implants were retrospectively evaluated. The obtained data were divided into two groups based on the type of auditory device used by the child—hearing aid or cochlear implant.

The inclusion criteria comprised families of up to 5 members, parents of children using hearing aids bilaterally or monolateral cochlear implants at the first application of the device; all children considered were those rehabilitated with a device (hearing aid or cochlear implant) that provided effective auditory stimulation within 36 months of age. Exclusion criteria included couples with children who had in their families children with other issues such as syndromes or medical conditions, children with a single parent, and the presence of economic problems or other familial issues that could compromise the adequate development of the child’s auditory and linguistic abilities. Parents included in the study reported no additional family conditions that could be considered sources of stress.

The study was conducted in accordance with the Declaration of Helsinki and approved by the local Ethics Committee of IRCCS Oasi Maria SS. (Prot, n. v.1.0 del 18 September 2024) for studies involving humans.

### 2.2. Procedure

Eighteen months after the initial activation of the devices, parents were asked to complete two self-assessment questionnaires—the LittlEARS Auditory Questionnaire (LEAQ) [13,14,15,16] and the Parental Stress Scale (PSS) [17]. The Parental Stress Scale assesses the level of stress perceived by parents in their relationship with their children. It consists of 18 items that focus on three main themes: ‘Positive emotional benefits’, ‘Sense of personal enrichment and fulfilment’ and ‘Negative aspects’. Parents respond to each item based on their own experience of their relationship with their children, using a five-point scale (1 = ‘strongly disagree’, 5 = ‘strongly agree’). Higher scores indicate higher levels of parental stress. The LEAQ is an instrument consisting of 35 questions that investigate various aspects of a child’s auditory perception and response to sound stimuli, from a few weeks of age up to about two years. The questionnaire monitors the progress of children with hearing impairment or who use amplification devices such as cochlear implants or hearing aids. LEAQ and PSS were translated into Italian by language experts.

The questionnaires were administered to the parents by an experienced audiologist who recorded their responses. The mother and father jointly completed a single questionnaire, reflecting on their child’s situation. Additionally, information was collected regarding the presence of peripartum issues (including preterm birth) or associated conditions, congenital hearing loss, the total number of children in the family, and the number of children with hearing loss in the family. By congenital hearing loss, we refer to children who did not pass the newborn hearing screening with otoacoustic emissions. All children who passed the newborn screening but whose hearing loss was diagnosed later were classified as having non-congenital hearing loss.

When assessing auditory abilities using the LEAQ, the “hearing age” rather than chronological age was considered, as the presence of hearing loss prevents adequate auditory development. Additionally, for children with cochlear implants, the period of fitting with conventional hearing aids was not considered since these devices often fail to provide sufficient auditory stimulation or, as is frequently the case, are not used by the children at all [16]. In reference to this aspect, we considered the number of months in which the effective auditory stimulation started. We mean, for children with hearing aids, the time in months from the birth to the first hearing aid fitting; for children with cochlear implants, we mean the time in months from birth to the implant’s first activation.

In the study, all children demonstrated high compliance with the device (CI or HA) usage, maintaining usage for above 12 h daily.

The choice of the 18-month period was made considering the authors’ personal experience at their audiology center, where they believe that 18 months is a sufficient timeframe to achieve an adequate auditory outcome. Furthermore, this observation is supported by Sunita (2024) in her article, which evaluated speech and auditory outcomes in children who underwent cochlear implantation before the age of six, demonstrating a statistically significant difference in auditory performance at 18 months [18]. Moreover, most studies evaluating auditory performance in children who have undergone cochlear implantation assess outcomes after a minimum of 12 months [19].

### 2.3. Statistical Analysis

The numerical data were expressed as median and interquartile range (Q1–Q3), and the categorical variables as absolute frequencies and percentages.

A non-parametric approach was used, since variables were not normally distributed, as verified by the Kolmogorov-Smirnov test, except for the PSS score.

The Chi-square test was performed to evaluate the presence of statistically significant differences between the categorical variables: device used and presence of peripartum issues or other associated conditions, and congenital hearing loss.

The Mann–Whitney test was used to assess significant differences between the two groups in terms of the number of months to diagnosis, months to effective auditory stimulation, age of the children at the time of the survey, total number of children and children with hearing loss, LEAQ and PSS scores.

The Spearman correlation test was conducted to evaluate the interdependence between the age of the children at the time of the survey and the LEAQ and PSS scores.

Univariate and multivariate logistic regression analyses were conducted to assess the relationship between the LEAQ score and various factors, including the type of device used, time to diagnosis (in months), time to effective auditory stimulation (in months), presence of peripartum complications or other associated conditions, congenital hearing loss, and total number of children. Similarly, univariate and multivariate linear regression analyses were performed to examine the association between the PSS score and the same variables. In the logistic regression model, our attention was focused on the dichotomy “Pathological or Not” for the LEAQ outcome. Specifically, patients with a LEAQ score of 30 or lower were classified as pathological, while those with a score above 30 were considered non-pathological. It is important to note that the value of 30 was not arbitrarily chosen by the authors; rather, it represents the average score corresponding to auditory milestones typically achieved at 18 months of amplification [14].

Statistical analyses were performed using SPSS 27.0 for Windows.

A *p*-value lower than 0.05 was considered statistically significant.

## 3. Results

The sample consisted of 42 pairs of parents of children with hearing aids (42.9%) or cochlear implants (57.1%). The median age at diagnosis was 6 months for the hearing aid group and 9 months for the cochlear implant group; the median ages for the initiation of effective auditory stimulation are 10 months and 15.5 months for children with hearing aids and cochlear implants, respectively (Table 1).

The Mann–Whitney test revealed a statistically significant difference when comparing the months to the initiation of effective stimulation (*p* = 0.026), the age of the children at the time of the survey (*p* = 0.024) and the PSS score (*p* = 0.029) between groups (Table 1).

The chi square test showed no statistically significant differences in the categorical variables related to the type of device used, the presence of congenital hearing loss, and peripartum issues or associated conditions (Table 2 and Table 3).

Univariate logistic regression for the LEAQ showed a statistically significant difference with changes in the number of months to effective stimulation (*p* = 0.007 with an odds ratio of 1.176) and the ages of the children at the time of the survey (*p* = 0.014 with and odds ratio of 1.162). This significance was also observed in the multivariate analysis (*p* = 0.025 with an odds ratio of 1.510 for the month to effective auditory stimulation and *p* = 0.012 with an odds ratio of 1.166 for the age of the children at the time of the survey) (Table 4).

**Figure 1:**
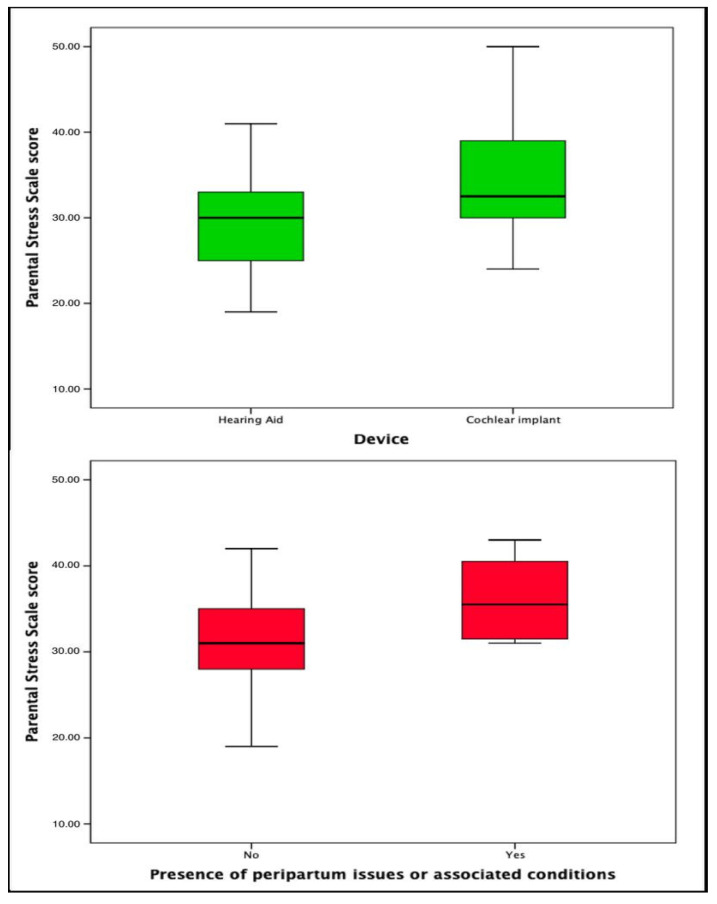
Boxplot representing Parental Stress Scale scores among hearing aid vs. cochlear implant users and in children with peripartum issues or associated conditions.

Univariate linear regression analysis for the PSS revealed significance in terms of the type of device (*p* = 0.020), the months to effective stimulation (*p* = 0.017), the age of the children at the time of the survey (*p* = 0.021), the presence of peripartum issues or associated conditions (*p* = 0.045), and the number of children (*p* = 0.043); similar results were obtained in the multivariate analysis (*p* = 0.044, *p* = 0.045, *p* = 0.038, *p* = 0.034, and *p* = 0.047, respectively) (Table 5) (Figure 1).

In Table 6, for the multivariable linear regression model, the R^2^ and adjusted R^2^ measures are reported to evaluate the explanatory power of all covariates inserted into the models.

In addition, for the multivariable binary logistic regression model, the Nagelkerke, Cox and Snell and McFadden R-square measures are reported to provide useful information regarding the goodness of the model’s fit to the data.

To estimate the goodness of fit of the linear regression model, we reported R^2^ and the adjusted R^2^. More specifically, the R^2^ measures the percentage of the variability of the dependent variable explained by the model; it represents the percentage by which the variability of the model errors is reduced relative to the variance of the dependent variable. The adjusted R-square is used in multiple regression to assess the degree of strength or effectiveness of the independent variables in explaining the dependent variable; the adjusted R^2^ expresses what percentage of the variation in the dependent variable is explained collectively by all the independent variables. Applied to our data, they are quite high (both higher than 0.75), denoting a good degree of adaptation of the linear model to the data.

To estimate the goodness of fit of the multivariable logistic regression model to the data, all three R^2^ measure (Nagelkerke, Cox and Snell and McFadden equal to 0.892, 0.809 and 0.797, respectively) provide information about an adequate degree of fit of the binary logistic model to the data. Furthermore, the Hosmer and Lemeshow test was also performed, confirming the adequacy of the model’s fit to the empirical data; in fact, being statistically not significant, it guarantees that the estimates provided by the multivariable model do not significantly differ from the observations.

A Spearman’s rho correlation analysis was conducted between the age of the children at the time of the survey and the LEAQ score, and the age of the children at the time of the survey and PSS scores, demonstrating, respectively, a correlation coefficient of 0.430 with a significance of *p* = 0.004 and a correlation coefficient of −0.317 with a significance of *p* = 0.041.

## 4. Discussion

Childhood hearing loss is a significant public health issue affecting millions of children worldwide, with profound implications for their development and quality of life. Untreated hearing loss can lead to a range of negative consequences, including reduced educational attainment, limited employment opportunities, and psychological distress [1,2,3,20,21,22].

Effective intervention for childhood hearing loss requires a multidisciplinary approach involving healthcare professionals, educators, and families. Tailored programs incorporating amplification devices, auditory–verbal therapy, educational support, and assistive technologies are essential for fostering language development, social integration, and academic success in children with hearing loss [23,24].

The effect of hearing loss on the children’ family is equally significant. Parents of children who are deaf or hard of hearing face unique challenges, often experiencing heightened levels of stress, increased out-of-pocket expenses, and more frequent absences from work compared to other parents [25]. Difficulties in communication with their children and the heightened need for support and financial resources can further compound this stress [10,26,27].

The unique circumstances facing parents of hearing-impaired children, particularly their psychological distress, have been examined in several studies [11]. These studies have investigated either parents of children with hearing aids or parents of children with cochlear implants as distinct groups [11].

Taking these premises into consideration, a comparative study was conducted between children with hearing impairments rehabilitated with conventional hearing aids and children with cochlear implants. The assessment involved comparing auditory maturation, evaluated using the LEAQ, and the level of stress experienced by the parental couple (mother and father), assessed using the PSS questionnaire. The aim was to investigate the correlation between the child’s level of maturation (and therefore auditory capacity) and parental stress.

Burger et al. evaluated the parental psychological state in parents of children with hearing loss who were using either hearing aids or cochlear implants at two different time points: after the definitive diagnosis and following the first fitting [12]. The authors concluded that the period immediately following the diagnosis is the most impactful in terms of parental stress, and that this stress tends to decrease after the first fitting [12]. Spahn et al. arrived at the same conclusion in their study [11].

In our study, we retrospectively analyzed data from children using conventional hearing aids and cochlear implants. No statistically significant difference was found in the number of months to hearing loss diagnosis between the two groups. This result is expected, given the performance of thorough neonatal screening, which allows for timely diagnosis. However, a significant difference was observed in the months to effective auditory stimulation, which was higher for cochlear implants. Comparing LEAQ and PSS scores, the only significant difference noted was in parental stress levels, which were higher for parents of children with cochlear implants. This finding aligns with those in the existing literature. No significant difference was noted in LEAQ scores, indicating comparable auditory performances in children from both groups.

Regarding the higher level of parental stress observed in the group of parents of children with cochlear implants, we believe this could be attributed to various factors. In particular, the stress may stem from the psychological challenges faced by parents during the pre-implantation process and surgery, which could make them more skeptical about the auditory recovery of their child. Additionally, the fitting process and management of cochlear implants are more complex compared to conventional hearing aids, especially concerning the mapping phases and rehabilitation, which may, to varying degrees, discourage parents.

Univariate and multivariate logistic regression analysis revealed an Odds Ratio greater than 1, with a statistically significant relationship between LEAQ scores and months to effective stimulation and age of the children at the time of the survey. It is well established that the timing of effective stimulation, and consequently the age of the children at the time of the survey, significantly impacts a child’s auditory performance. Univariate and multivariate linear regression analysis for PSS showed the significant influence of the type of device used (HA or CI), months to effective stimulation, age of the children at the time of the survey, presence of perinatal issues or associated syndromes, and the total number of children. Multivariate linear analysis confirmed these results, indicating higher stress levels for parents of children with cochlear implants. As previously described, the diagnostic and therapeutic journey related to cochlear implantation is longer and more complex than that related to conventional hearing aids, contributing to increased parental stress. Additionally, the presence of peripartum issues or associated syndromes requires more attention from caregivers, and the number of children to care for adds further concern.

Moreover, a correlation study was conducted between the age of the children at the time of the survey and the LEAQ and PSS scores. A significant positive correlation was found between the age of the children at the time of the survey and the LEAQ score, demonstrating that, with increasing age, children’s auditory performance improves. In contrast, the age of the children at the time of the survey and the PSS score were negatively and significantly correlated, indicating lower parental stress as the children’s age increases. These findings are in line with the expected results.

Our study aims to further highlight the role of parents in managing children with hearing loss. Additionally, we investigated the correlation between auditory recovery and parental stress. While we acknowledge that similar studies have been conducted, our research group aims to emphasize the use of simple and quick tools, such as the PSS questionnaire we used, in assessing parental stress during the rehabilitation process of these children, especially for those with poor functional outcomes. Although these outcomes depend on various factors, the role of parents in achieving functional auditory results has been widely described. Therefore, in children with poor auditory recovery, interventions should address the causes leading to poor functional results, including parental involvement. In recent years, various studies have been published on this topic [9,28,29,30].

The LEAQ, on the other hand, is an effective tool for evaluating the auditory recovery of these patients over time, as it can be easily completed by parents throughout the rehabilitation process. We acknowledge that more objective tools for assessing auditory recovery in hearing-impaired children should be developed, and the use of self-assessment questionnaires in clinical practice can only reinforce the information available on the functional outcomes obtained. For these reasons, we believe that the tools used in this study can be valuable in daily clinical practice.

Clinically, parental stress is often overlooked during the rehabilitation of children with hearing impairments, regardless of whether they use hearing aids or cochlear implants. The stress of parents of children with cochlear implants is influenced by several factors that go beyond medical and rehabilitation challenges [10]. Socio-economic status, availability of support networks, and pre-existing conditions of the parents contribute significantly to the stress experienced during the cochlear implantation process. Socioeconomic status plays a key role in determining access to healthcare resources and the ability to manage the financial demands of cochlear implantation, including surgery, device maintenance and rehabilitation services. Families with limited financial resources may experience greater stress due to these costs, which may be compounded by reduced access to specialized support services, unlike families with higher socioeconomic status. The presence of strong support networks, such as family, friends and community resources, is another important factor that can decrease parental stress by providing emotional reassurance and assistance. Conversely, the absence of such networks often results in a sense of isolation, intensifying stress. The literature has shown that perceived social support is inversely related to parental stress, emphasizing the importance of promoting support systems [31]. Pre-existing psychological conditions in parents, such as anxiety or depression, may increase stress during cochlear implantation, as these conditions may reduce coping skills and increase psychological vulnerability [32]. The importance of counseling for parents involved in the diagnostic and rehabilitative process cannot be understated. Addressing these factors through personalized interventions, such as financial support programs and peer networking, could significantly reduce stress and improve outcomes for both parents and children undergoing cochlear implantation.

In light of the above, it is important to clarify a key aspect. The two auditory rehabilitation systems—hearing aids and cochlear implants—utilize different modes of stimulation. At first glance, the auditory and functional outcomes of these patients might not seem directly comparable or assessable simultaneously. However, the ultimate goal of auditory rehabilitation, whether through hearing aids or cochlear implants, is to ensure linguistic and listening skills that are equivalent (or nearly equivalent) to and aligned with those of normal-hearing children of the same age. Furthermore, the focus of this article is not to determine which of the two systems (hearing aids or cochlear implants) is better, as it is well-known that the indications for using one over the other are different.

Our study, although seemingly intuitive, provides specific LEAQ and PSS score benchmarks for comparison, and paves the way for future research to integrate the combination of LEAQ and PSS into the rehabilitative journey of these children. While both instruments have been independently validated for clinical use, the utility and combined roles of these measures still require rigorous validation. Furthermore, having assessed baseline conditions in “stress-free parents”, a longitudinal study examining parents facing other stressors would be valuable. Regular assessments, such as every 3 or 6 months, would provide insights into the evolution of parent stress levels and their impacts on hearing outcomes.

However, we must specify that the study has limitations and that the data presented are only an excerpt of the practice at our center. First, the study is based on a small number of patients; conducting a study with a larger or multicentric sample would be appropriate to confirm the results obtained. Second, the study assumes that stress depends solely on the condition of the children’s hearing loss; however, other stressors could be present within the couples, thus influencing the results. Third, the data collected are based only on the eighteenth month, even though repeatedly administering the questionnaires at more defined intervals could be more useful in evaluating the progress of auditory recovery and parental stress.

## 5. Conclusions

In our comparative study, we found that parents of children with cochlear implants report higher stress levels than those with children using conventional hearing aids. However, auditory recovery, as measured by the LEAQ, was similar for both groups at 18 months of hearing age. Importantly, improved auditory performance in children was associated with reduced parental stress.

This research underscores the potential utility of LEAQ and PSS in clinical settings. The PSS is a quick and simple tool for assessing parental stress, while the LEAQ effectively tracks auditory recovery over time. These tools can help clinicians better understand and support both the auditory development of children and the well-being of their parents.

Although our study has limitations, the results hint at the potential use of LEAQ and PSS for monitoring auditory outcomes and parental stress, thereby improving support for families during rehabilitation. Further research with larger samples is needed to confirm these results and refine the approach to supporting families with hearing-impaired children. As a future perspective, it could be valuable to evaluate parental stress and auditory recovery in patients with delayed cochlear implantation during childhood, perinatal problems, inner ear anomalies, hearing aid nonuse or short-term use, comorbid disorders, long-term deafness, and intraoperative complications.

## Figures and Tables

**Table 1:** Study population numerical variables: median, 25th quartile, 75th quartile and comparison between used devices (cochlear implant vs. hearing aid).

	Cochlear Implant			Hearing Aid			*p*-Value
	25th	Median	75th	25th	Median	75th	
Months to diagnosis	6.00	6.00	16.50	6.00	9.00	18.50	0.241
Months to effective auditory stimulation	13.00	15.50	27.75	7.00	10.00	19.50	0.026 *
Age of the children at the survey	31.00	33.50	43.75	25.00	28.00	37.50	0.024 *
Total number of children	1.25	2.00	3.00	1.00	2.00	2.00	0.188
Children with hearing loss in the family	1.00	1.00	1.00	1.00	1.00	1.00	0.155
LEAQ score	22.25	28.00	31.75	29.00	30.50	33.00	0.095
PSS score	29.50	32.50	39.50	24.75	30.00	33.50	0.029 *

* the significant *p*-value at 0.05 significance level. LEAQ, LittlEARs Auditory Questionnaire; PSS, Parental Stress Scale.

**Table 2:** Contingency table and comparison between used device (cochlear implant vs. hearing aid) and presence of peripartum issues or associated conditions.

Presence of Peripartum Issues or Associated Conditions	Cochlear Implant N (%)	Hearing Aid N (%)	Total N (%)
No	14 (77.8%)	20 (83.3%)	34 (81.0%)
Yes	4 (22.2%)	4 (16.7%)	8 (19.0%)
Total	18 (100%)	24 (100%)	42 (100%)
Pearson chi-square test			*p*-value
			0.650

**Table 3:** Contingency table and comparison between used devices (cochlear implant vs. hearing aid) and congenital hearing loss.

Congenital Hearing Loss	Cochlear Implant N (%)	Hearing Aid N (%)	Total N (%)
No	4 (22.2%)	7 (29.2%)	11 (26.2%)
Yes	14 (77.8%)	17 (70.8%)	31 (73.8%)
Total	18 (100%)	24 (100%)	42 (100%)
Pearson chi-square test			*p*-value
			0.612

**Table 4:** Logistic regression.

	Univariable			Multivariable		
Variables	OR	95% CI	*p*-Value	OR	95% CI	*p*-Value
Device	2.000	0.570–7.013	0.279	0.164	0.009–2.990	0.223
Number of months to diagnosis	1.078	0.988–1.176	0.092	0.733	0.515–1.043	0.085
Months to effective auditory stimulation	1.176	1.045–1.322	0.007 *	1.510	1.061–2.250	0.025 *
Age of the children at the survey	1.162	1,018–1,230	0.014 *	1.166	1.034–1.316	0.012 *
Peripartum issues or other associated conditions	1.167	0.239–5.698	0.849	0.496	0.045–5.510	0.568
Congenital hearing loss	0.237	0.044–1.280	0.094	0.190	0.014–2.611	0.214
Number of children	0.940	0.436–2.026	0.874	0.609	0.190–1.954	0.405

* the significant *p*-value at 0.05 significance level.

**Table 5:** Linear regression.

	Univariable			Multivariable		
Variables	B	95% CI	*p*-Value	B	95% CI	*p*-Value
Device	4.694	0.771–8.617	0.020 *	4.097	0.129–8.106	0.044 *
Number of months to diagnosis	0.105	−0.98–0.308	0.301	−0.155	−0.621–0.311	0.503
Months to effective auditory stimulation	0.242	0.046–0.438	0.017 *	0.202	0.012–0.499	0.045 *
Age of the children at the survey	0.242	0.059–0.477	0.021 *	0.255	0.078–0.671	0.038 *
Peripartum issues or other associated conditions	5.007	0.038–10.053	0.045 *	5.310	0.442–10.882	0.034 *
Congenital hearing loss	−2.613	−7.266–2.040	0.263	−0.472	−5.884–4.940	0.860
Number of children	2.204	0.003–4.410	0.043 *	2.423	0.039–4.808	0.047 *

* the significant *p*-value at 0.05 significance level.

**Table 6:** Measures of adequacy and goodness of fit of multivariate models to data.

**Multivariable Linear Regression Model (PSS)**	
R^2^	0.812
Adjusted R^2^	0.791
**Multivariable Logistic Regression Model (LEAQ)**	
**Measure**	**Value**
Nagelkerke R^2^	0.892
Cox and Snell R^2^	0.809
McFadden R^2^	0.797
Hosmer–Lemeshow Test	*p*-value
5.945	0.654

LEAQ, LittlEARs Auditory Questionnaire; PSS, Parental Stress Scale.

## Data Availability

All data of the study are available on request to the corresponding author.

## References

[1] World Health Organization (2021). World Report on Hearing.

[2] Al-Ani R.M. (2023). Various Aspects of Hearing Loss in Newborns: A Narrative Review. World J. Clin. Pediatr..

[3] World Health Organization (2016). Childhood Hearing Loss: Strategies for Prevention and Care.

[4] World Health Organization (2021). Hearing Screening: Considerations for Implementation.

[5] Ching T.Y.C., Dillon H., Leigh G., Cupples L. (2017). Learning from the Longitudinal Outcomes of Children with Hearing Impairment (LOCHI) Study: Summary of 5-Year Findings and Implications. Int. J. Audiol..

[6] Dettman S.J., Pinder D., Briggs R.J.S., Dowell R.C., Leigh J.R. (2007). Communication Development in Children Who Receive the Cochlear Implant Younger than 12 Months: Risks versus Benefits. Ear Hear..

[7] Ching T.Y.C., Dillon H., Button L., Seeto M., Van Buynder P., Marnane V., Cupples L., Leigh G. (2017). Age at Intervention for Permanent Hearing Loss and 5-Year Language Outcomes. Pediatrics.

[8] Portelli D., Loteta S., Ciodaro F., Salvago P., Galletti C., Freni L., Alberti G. (2024). Functional Outcomes for Speech-in-Noise Intelligibility of NAL-NL2 and DSL v.5 Prescriptive Fitting Rules in Hearing Aid Users. Eur. Arch. Otorhinolaryngol..

[9] Anmyr L., Larsson K., Olsson M. (2016). Parents’ Stress and Coping Related to Children’s Use of a Cochlear Implant: A Qualitative Study. J. Soc. Work Disabil. Rehabil..

[10] Zaidman-Zait A. (2008). Everyday Problems and Stress Faced by Parents of Children with Cochlear Implants. Rehabil. Psychol..

[11] Spahn C., Richter B., Burger T., Löhle E., Wirsching M. (2003). A Comparison between Parents of Children with Cochlear Implants and Parents of Children with Hearing Aids Regarding Parental Distress and Treatment Expectations. Int. J. Pediatr. Otorhinolaryngol..

[12] Burger T., Spahn C., Richter B., Eissele S., Lohle E., Bengel J. (2005). Parental Distress: The Initial Phase of Hearing Aid and Cochlear Implant Fitting. Am. Ann. Deaf.

[13] Schaefer K., Coninx F., Fischbach T. (2019). LittlEARS Auditory Questionnaire as an Infant Hearing Screening in Germany after the Newborn Hearing Screening. Int. J. Audiol..

[14] Coninx F., Weichbold V., Tsiakpini L., Autrique E., Bescond G., Tamas L., Compernol A., Georgescu M., Koroleva I., Le Maner-Idrissi G. (2009). Validation of the LittlEARS^®^ Auditory Questionnaire in Children with Normal Hearing. Int. J. Pediatr. Otorhinolaryngol..

[15] Visram A.S., Purdy S.C., Kelly J., Munro K.J. (2022). Longitudinal Assessment of Listening Skills in UK Infants with Hearing Aids Using the LittlEARS^®^ Auditory Questionnaire. Int. J. Audiol..

[16] Obrycka A., Lorens A., Padilla García J.-L., Piotrowska A., Skarzynski H. (2017). Validation of the LittlEARS Auditory Questionnaire in Cochlear Implanted Infants and Toddlers. Int. J. Pediatr. Otorhinolaryngol..

[17] Berry J.O., Jones W.H. (1995). The Parental Stress Scale: Initial Psychometric Evidence. J. Soc. Pers. Relatsh..

[18] Sunita D. (2024). Prediction of Factors Affecting Speech and Auditory Outcomes of Cochlear Implantation in Pre-Lingual Deaf Children: A Cross-Sectional Study. Am. J. Otolaryngol. Head Neck Surg..

[19] Wu S.S., Sbeih F., Anne S., Cohen M.S., Schwartz S., Liu Y.C., Appachi S. (2023). Auditory Outcomes in Children Who Undergo Cochlear Implantation Before 12 Months of Age: A Systematic Review. Otolaryngol. Head Neck Surg..

[20] Stevenson J., McCann D., Watkin P., Worsfold S., Kennedy C. (2009). The Relationship between Language Development and Behaviour Problems in Children with Hearing Loss. J. Child Psychol. Psychiatry.

[21] Mason A., Mason M. (2007). Psychologic Impact of Deafness on the Child and Adolescent. Prim. Care Clin. Off. Pract..

[22] Alberti G., Portelli D., Galletti C. (2023). Healthcare Professionals and Noise-Generating Tools: Challenging Assumptions about Hearing Loss Risk. Int. J. Environ. Res. Public Health.

[23] Yoshinaga-Itano C., Sedey A.L., Wiggin M., Chung W. (2017). Early Hearing Detection and Vocabulary of Children with Hearing Loss. Pediatrics.

[24] Joint Committee on Infant Hearing (2019). Year 2019 Position Statement: Principles and Guidelines for Early Hearing Detection and Intervention Programs. J. Early Hear. Detect. Interv..

[25] Barton G.R., Stacey P.C., Fortnum H.M., Summerfield A.Q. (2006). Hearing-Impaired Children in the United Kingdom, IV: Cost-Effectiveness of Pediatric Cochlear Implantation. Ear Hear..

[26] Wood Jackson C., Turnbull A. (2004). Impact of Deafness on Family Life. Top. Early Child. Spec. Educ..

[27] Zaidman-Zait A., Most T., Tarrasch R., Haddad-eid E., Brand D. (2016). The Impact of Childhood Hearing Loss on the Family: Mothers’ and Fathers’ Stress and Coping Resources. J. Deaf Stud. Deaf Educ..

[28] Sparreboom M., Leeuw A.R., Snik A.F.M., Mylanus E.A.M. (2012). Sequential Bilateral Cochlear Implantation in Children: Parents’ Perspective and Device Use. Int. J. Pediatr. Otorhinolaryngol..

[29] Hyde M., Punch R., Komesaroff L. (2010). A Comparison of the Anticipated Benefits and Received Outcomes of Pediatric Cochlear Implantation: Parental Perspectives. Am. Ann. Deaf.

[30] Sach T.H., Whynes D.K. (2005). Paediatric Cochlear Implantation: The Views of Parents. Int. J. Audiol..

[31] Zaidman-Zait A., Curle D., Jamieson J.R. (2023). Health-related quality of life among mothers of children with cochlear implants with and without developmental disabilities. Res. Dev. Disabil..

[32] Zhang X., Xie J., Wu W., Cao L., Jiang Z., Li Z., Li Y. (2024). The mediation effect of mental resilience between stress and coping style among parents of children with cochlear implants: Cross-sectional study. J. Pediatr. Nurs..

